# The debate on the earthquake magnitude correlations: a meta-analysis

**DOI:** 10.1038/s41598-022-25276-1

**Published:** 2022-11-30

**Authors:** Giuseppe Petrillo, Jiancang Zhuang

**Affiliations:** The Institute of Statistical Mathematics, Research Organization of Information and Systems, Tokyo, Japan

**Keywords:** Seismology, Statistics

## Abstract

Among the most important questions that await an answer in seismology, perhaps one is whether there is a correlation between the magnitudes of two successive seismic events. The answer to this question is considered of fundamental importance given the potential effect in forecasting models, such as Epidemic Type Aftershock Sequence models. After a meta-analysis of 29 papers, we speculate that given the lack of studies carried out with realistic physical models and given the possible bias due to the lack of events recorded in the experimental seismic catalogs, important improvements are necessary on both fronts to be sure to provide a statistically relevant answer.

## Introduction

The correlation between variables is closely linked to the concept of forecast. In fact, correlations are useful for prediction, even when there is no known causal relationship between the two variables. Clearly, a better model is often possible if a causal mechanism can be determined. In fact, the search for correlations and possibly causal relationships between variables is a constant challenge for statisticians. The fields of applications are obviously the most diverse and range from economic fields such as applications in finance, marketing and risk management^1,2^ to more technical-scientific uses such as seismic forecasting^3–5^. In the earthquake occurrence, magnitude correlation has much older origins than is thought. In fact, this problem is closely related to seismic predictability. More precisely, from the hypothesis that the seismic process can be described as Self Origanized Critical (SOC)^6^ phenomena, an intrinsic unpredictability may be attributed to the seismic occurrence. However, strong opposition to this hypothesis has been made by the seismological community. In particular^7^ showed that the Southern California seismic catalog is not invariant under event reshuffling and therefore, with good confidence level, the theory that earthquakes are a SOC process could be discarded and could no longer be used as a bypass for avoid answering the question “can earthquakes be predicted?”. From this moment on, a back and forth of comments and articles on the subject began. In particular, just as the temporal clustering of earthquakes has been confirmed thanks to Omori observation^8^, the spatial clustering is now well accepted^9^. In practice, all the statistical laws that are universally accepted have been implemented in the so-called Epidemic Type Aftershock Sequence (ETAS) model, which represents the gold standard for forecasting and seismic clustering^10–16^. The magnitude correlation is discussed, not just to understand the physics of the process, but also to understand whether further improvements to the ETAS model are necessary, i.e., implement the phenomenon of clustering in magnitude. It is important to underline how a modification of an important forecasting tool such as the ETAS model can be of vital importance to obtain even a small improvement on a probable seismic forecast.

However, the study of correlations in seismology is not simple and immediate. In this case the problem is complicated by the intrinsic incompleteness of the experimental catalogs: after the occurrence of a large earthquake, not all events can be recorded due to the overlapping of coda waves. For this reason, the events recorded in the seismic catalogs are not all those that actually occur^17–20^. By studying these catalogs, a statistically significant correlation is observed between the magnitudes of subsequent events. The overwhelming majority of the debate focuses precisely on this point. In fact, the presence of these clear correlations can be a spurious effect due to the incompleteness of the catalog. In other words, the criticism of the existence of correlations is related to the fact that, if the catalog were complete, the observed magnitudes would be independent.

**Figure 1:**
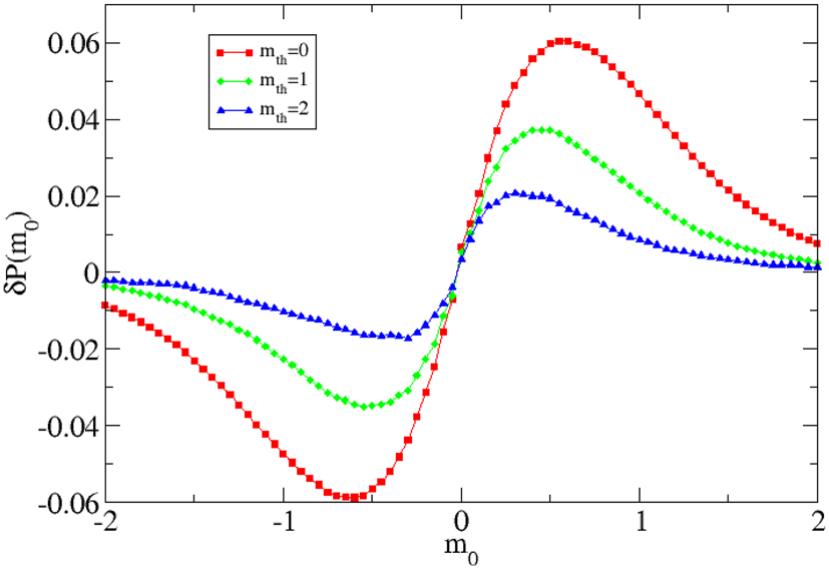
$\delta P$ as a function of $m_0$ for the southern California relocated catalog. Different symbols and colors indicate different $m_{th}$ considered.

For example, in Fig. 1, from^21^, the quantity $\delta P(m_0)$ for different magnitude threshold $m_{th}$ is plotted. The distribution $\delta P(m_0)$, as defined in^22^, represents the difference between the probability $P(\Delta m < m_0)$ and $P(\Delta m^* < m_0)$, namely the probability to observe the number of couples of subsequent events with a magnitude difference $< m_0$. Here $\Delta m$ is the magnitude difference between the subsequent events and $m^*$ represents the magnitude difference made with random chosen event inside the catalog. Therefore the magnitude difference computed with $m^*$ is uncorrelated by definition. In absence of correlations $\delta P(m_0)$ should not significantly deviate from 0 for all $m_0$. In Fig. 1, one can observe that reducing $m_{th}$ the deviation from 0 become more important and the fact that the catalog considered becomes more incomplete for smaller values of $m_{th}$ suggests that this trend is a direct consequence of the catalog incompleteness.

In order to address the short-term aftershock incompleteness, physical models (Olami-Feder-Christensen (OFC)-like) were used to produce complete synthetic seismic catalogs^23^. In addition, dynamical scaling topics were also proposed^24,25^ to explain the correlation between magnitude. In these studies the main assumption is that the magnitude difference between two event *i* and *j* fixes a characteristic time, $\tau _{ij}$, so that the conditional rate is magnitude independent when time is rescaled by $\tau _{ij}$. In practice $\rho (m_i(t_i)|m_j(t_j)) = F(\frac{t_i-t_j}{\tau })$ . During the last years a further branching of the studies has been observed, some of which implement an ETAS model with the correlations between magnitude. They also test if this modified ETAS model matches better the experimental data than the original ones, while others that try to understand if there is clustering between the magnitudes with alternative methods.

## Results

### Analysis and classification

In Table 1 we present a list of 29 papers published between the years 1989 and 2022 to demonstrate that the answer to the question “Do earthquakes in a cluster presents magnitude correlation?” has not yet been provided and further studies are needed. The dataset acronyms used in the studies are shown in Table 2.

Starting from the 1989 study by Bak and Tang^6^, a series of articles, both statistical and heuristic, and also based on physical models (i.e. spring block models) have been published to give a contribution to the understanding of the phenomenon of correlation between magnitude. This study is also known to have paved the way for statistical mechanics to study the phenomenon of seismic events. In addition to this, it builds a bridge between the community of physicists and of seismologists.

**Table 1:** Scientific papers considered in the meta-analysis.

Number	Year	Reference	Data	Method	Correlations	Author
1	1989	^6^	Numerical	Heuristic	No	Bak & Tang
2	2002	^26^	SCSN	Statistics	No	Christensen et al.
3	2004	^27^	CMT,NEIC,CNSS	Statistics	No	Felzer et al.
4	2004	^7^	SCSN	Heuristic	Yes	Yang et al.
5	2005	^28^	SCSN	Heuristic	No	Corral
6	2006	^29^	ANSS	Statistics	No	Helmstetter et al.
7	2006	^30^	NEIC-PDE	Statistics	No	Corral
8	2007	^24^	ANSS	Statistics	Yes	Lippiello et al.
9	2007	^25^	ANSS	Statistics	Yes	Lippiello et al.
10	2007	^31^	NCEDC	Physics	No	Caruso et al.
11	2008	^22^	NCEDC	Statistics	Yes	Lippiello et al.
12	2009	^32^	SCEC,JMAEC	Statistics	Yes	Sarlis et al.
13	2009	^33^	NCEDC	Statistics	Yes	Lippiello et al.
14	2010	^34^	CMT	Statistics	No	Yoder et al.
15	2010	^35^	CMT	Statistics	No	Aalsburg et al.
16	2011	^36^	Numerical	Physics	No	Zhang et al.
17	2011	^37^	SCEC, CMT	Statistics	Yes	Sarlis
18	2011	^21^	^38^	Statistics	No	Davidsen
19	2012	^39^	^40^	Statistics	Yes	Lippiello et al.
20	2012	^41^	^42^	Statistics	No	Davidsen et al.
21	2013	^23^	Numerical	Physics	Yes	Lippiello et al.
22	2013	^43^	CMT	Statistics	Yes	Nichols et al.
23	2013	^44^	Numerical	Physics	No	Shcherbakov et al.
24	2016	^45^	ISIDE,^46^	Statistics	Yes	Spassiani et al.
25	2016	^47^	Numerical	Statistics	Yes	Spassiani et al.
26	2018	^48^	TABOO,SOCAL,JMA	Statistics	No	Stallone et al.
27	2019	^49^	SCSN	Statistics	No	Zambrano
28	2019	^50^	CMT	Statistics	Yes	Nandan et al.
29	2022	^51^	ANSS	Statistics	Yes	Nandan et al.

**Table 2:** Acronym for the seismic catalogs considered in the studies.

Acronym	Catalog
CMT	Global Centroid Moment Tensor
NEIC	National Earthquake Information Center
CNSS	California Council of the National Seismic System
SCSN	Southern California Seismographic Network
ANSS	Advanced National Seismic System
PDE	Preliminary Determination of Epicenter
JMAEC	Japanese Meteorological Agency Earthquake Catalog
SCEC	Southern California Earthquake Catalog
NCEDC	Northern California Earthquake Data Center
ISIDE	Italian Seismological Instrumental and Parametric Data-Base
TABOO	Italian Alto Tiberina Near Fault Observatory earthquake catalogue
SOCAL	Southern California Relocated Catalog
JMA	Japanese Meteorological Agency

However, analyzing all the studies considered to date, we find that 51% of them reject the hypothesis of magnitude correlation, while the 49%, observe a non-zero correlation.

**Figure 2:**
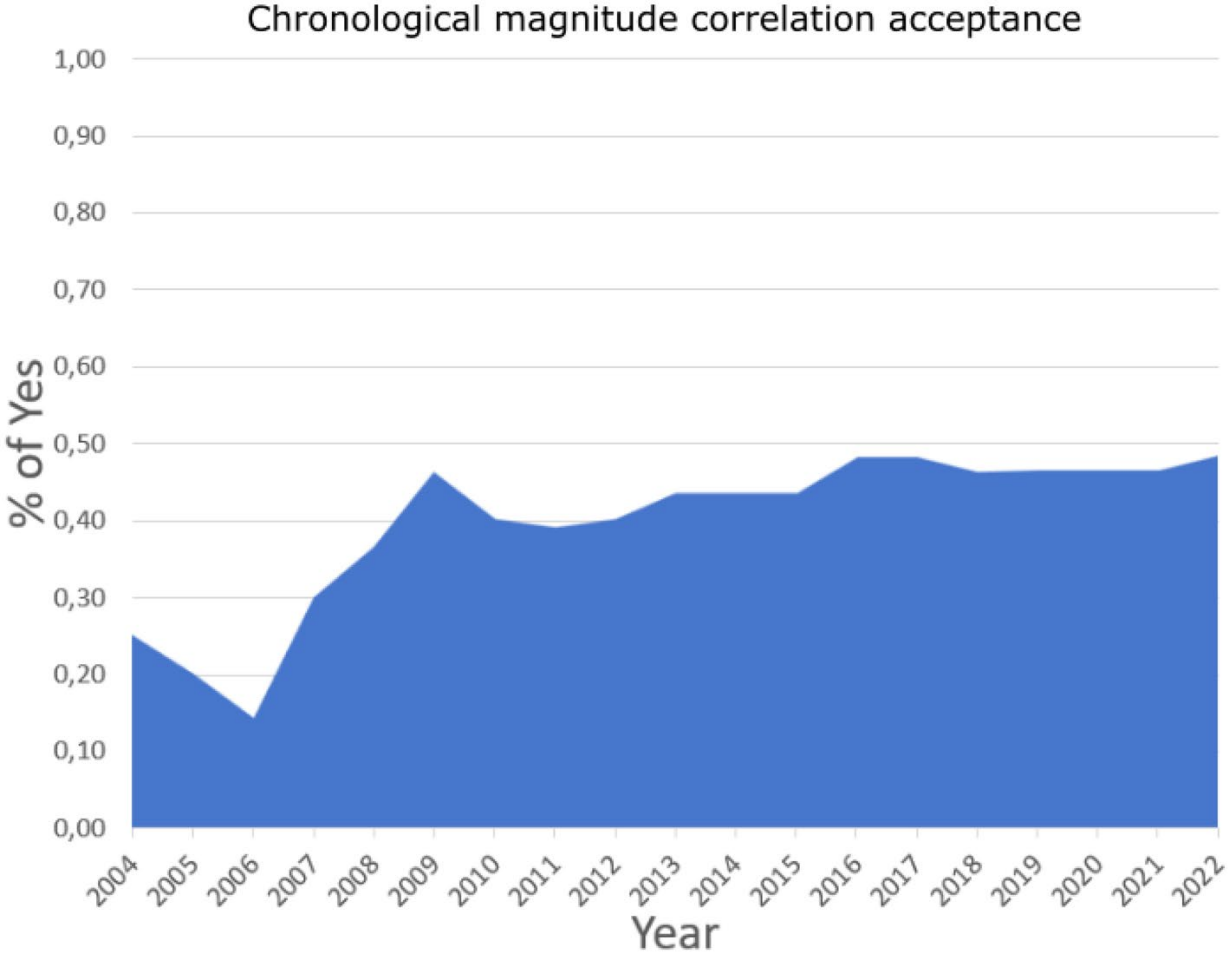
Time course of the percentage of studies that accept the existence of the correlation between magnitude.

**Figure 3:**
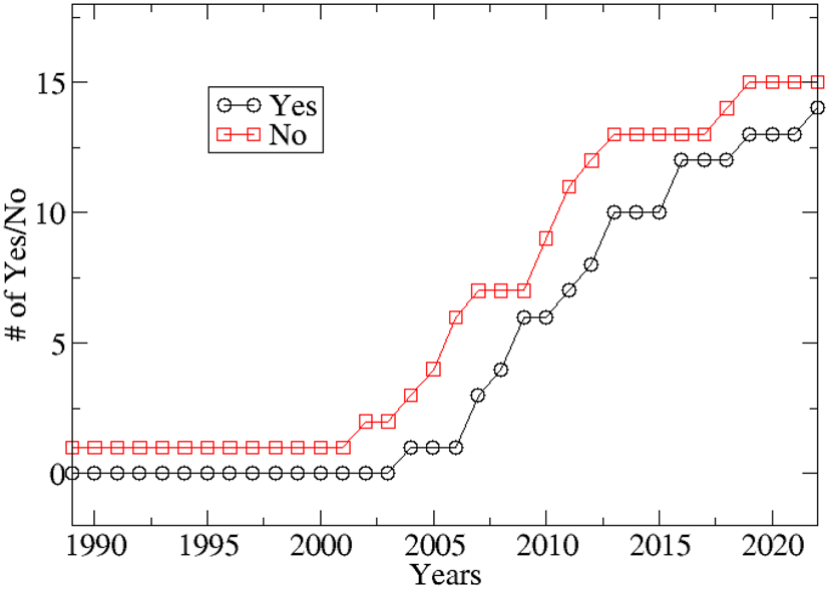
Cumulative number of acceptance of the correlation hypothesis (yes) and rejection (no) of the correlation hypothesis over the years.

Investigating the studies chronologically (Fig. 2), we note that the percentage of scientists that accept the magnitude correlation hypothesis it slightly increased over the time between the 2004 and 2009. While, from 2010 on-wards, a substantially constant trend between 40% and 50% is observed, indicating that the debate is still ongoing, and that an answer has not yet been universally accepted by the entire seismological community. This is also confirmed by observing the cumulative number of acceptance and rejection as a function of time of the correlation hypothesis in Fig. 3. In fact, in recent years, the rate of affirmative and negative replies seems to be comparable.

In Fig. 5, studies are separated by type of approach. We observe that the majority of published papers are based on statistical studies. For example, they analyse and test experimental data or verify whether an ETAS model, with magnitude correlations implemented, can fit those experimental data better than an ETAS model with independent magnitudes. It should be added that the statistical studies carried out on real data could even suffer of an imperfect distinction between clustered events (foreshocks, mainshock and aftershocks) and background events. In fact, even slightly mixing the two types of events could lead to a reduction of correlation evidence since, for the background activity, the independence between magnitudes seems to be universally accepted. In practice, even an improvement in declustering techniques could allow a step forward in the study of this phenomenon.

Due to the heterogeneity of the studies in terms of approach to the problem and in terms of experimental data used, we will proceed with a more detailed statistical analysis to make a fair comparison.

#### Sample size

**Figure 4:**
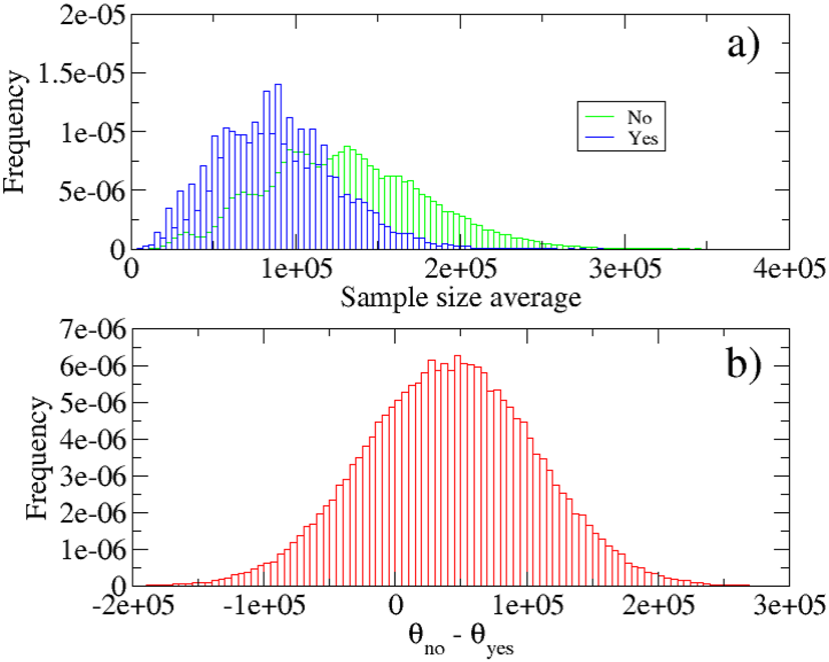
Results of the bootstrapping inference. (**a**) Bootstrap distributions of the number of events contained in the data-sets considered in the studies that has rejected the hypothesis of magnitude correlation (no) and accepted the hypothesis (yes). The mean was chosen as statistics. (**b**) The distribution of the difference $\theta _{no}-\theta _{yes}$.

In this section we will try to understand if the studies are homogeneous in terms of sample size or not. Given the poor statistics of papers, we use bootstrap as a re-sampling technique and compute the *p*-value. For the procedure we take into consideration only those papers in which the sample size is well declared or easily obtainable from an online seismic catalog. With this choice we get $s_{no}=(293405,101680,46937,46055,400000,11906,11906,452943,20000,32476)$ and $s_{yes}=(77955,9586,1000,4984,8502,85862,340000,100000,11535,320000)$ which represent the vectors containing the number of samples considered in the studies that rejected the hypothesis and do not reject the hypothesis, respectively. After re-sampling with replacement obtained numerically with $N=100,000$ realizations, it is possible to compute the average of each sample, $\theta _x=(\theta ^i_x)_{i=1,N}$, where the entries of *x* are *yes* or *no*, and calculate the distribution of the difference $\theta ^i_{no}-\theta ^i_{yes}$. The distributions of the means obtained by dividing the cases *yes* and *no* are shown in Fig. 4a, while the distribution of differences is shown in Fig. 4b. For the calculation of the *p*-value we employ a non-parametric version of a t-test, i.e., a permutation test. In practice we find a *p*-value counting all the results as or more extreme than the observed result (the difference of the original samples mean) and divide by *N*. We obtain $p=0.553$, which means that with a high level of confidence cannot reject the hypothesis that the number of samples, *yes* and *no*, are different. Therefore we conclude that the studies are homogeneous in terms of the number of samples used and a comparison can be made correctly.

#### Google Scholar citations

Proceeding similarly to the analysis on the size of the samples, in this section we show how not even in this case there is a preponderance of a preference side in the google scholar citations relative to the studies considered. In particular, after the bootstrapping inference procedure, the *p*-value found is $p = 0.45$. Therefore quantitatively, even in terms of popularity in the literature, the studies can be considered homogeneous.

#### Catalog incompleteness

To understand whether in the studies considered there is an association between the accepted hypothesis and the consideration or not of the short-term incompleteness in the instrumental catalog, we use the contingency tables and perform a $\chi ^2$-test. Comparison between the observed (Table 3a) and the expected distribution (Table 3b) distribution qualitatively suggests no association between the variables. More quantitatively, the calculation of $\chi ^2$ yields $\chi ^2=0.363$ which, for one degree of freedom, confirms the result with a high level of confidence. For this reason we observe no bias in the consideration or not in choosing the incompleteness and therefore the studies can be considered homogeneous also in terms of this variable.

**Table 3:** Contingency tables for the observed distribution (a) and expected distribution (b) related to the study on the association with the incompleteness of the experimental catalog.

**Observed distribution**			
	Yes	No	
Incompleteness	9	8	17
No Incompleteness	5	7	12
	14	15	29
**Expected distribution**			
	Yes	No	
Incompleteness	8.2	8.8	17
No incompleteness	5.8	6.2	12
	14	15	29

## Discussion

### Limitations

The purpose of a meta-analysis should be to combine the results in the literature and distill a result that gives a quantitatively and statistically accepted answer, all carried out on the basis of statistical tests. It is important to take into account that not only the single *p*-value is needed for the purposes of a fair comparison, namely, it is necessary to evaluate the importance of a result and not only the probability of it^52,53^. For this reason it is strongly suggested to study the Effect Size of the statistical result of each study carried out. For example, if Kendall’s Tau is available, one can easily convert it to study the Effect Size^54^. Unfortunately this is not possible to do in the present analysis due to a systematic lack of direct correlation tests. In this Section, we show the limitations of each type of study considered.

#### Heuristic approach

Although heuristic methods are not statistically useful for making inference, they pave the way for new ideas of development and attack on the problem under consideration. The case of the Bak and Tang^6^ paper is a striking example. The results of^7^ and^28^, both heuristics, cancel each other out and do not affect the analysis on the totality of the papers considered.

#### Physical approach

Although physical methods have the advantage of completely avoiding the short term aftershock incompleteness problem, they are subject to the limit of model imperfection. In fact, nowadays the development of these models proceed precisely in order to obtain a more faithful description of seismicity. In Table 1 There are 4 papers that use this type of approach, 3 of which claim an absence of correlations between magnitudes. Even if the number of results based on a physical model is very small, in this case we would be led to conclude that the absence of correlations between magnitudes is the most likely answer. Unfortunately, the greater presence of correlations between magnitudes is hypothesized to be present between clustered events (mainshock-aftershocks sequences) rather than between background events. Since the physical models used in the studies are OFC type, the answer is biased. In fact, it is well known that OFC-like model^55^ do not produce aftershocks. This could be consistent with the results in the literature that give a greater probability of rejecting the phenomenon of magnitude correlations.

#### Statistical approach

Statistical methods are the most numerous in this field of research. Unfortunately, only a few of these directly calculate correlations between magnitudes using a dataset. The others, on the other hand, define and use epidemic models with or without the implementation of the phenomenon of magnitude correlation and evaluate whether the model describes seismicity well.

Unfortunately, with the data obtained from the analyzed scientific studies it is not possible to reach a statistically significant conclusion. In order to carry out a test and to be able to carry out a fair comparison it is necessary, in addition to greater unanimity on the result, also to perform more direct studies on the correlation between magnitudes in order to be able to provide a statistically significant answer on the debate concerning the correlation between magnitude. We expect the result of this analysis to push both geophysics and statistical seismology communities to continue addressing the problem.

Our personal opinion is not to abandon the use of physical models just because they do not perfectly describe realistic seismicity but rather to continue the research towards ever more realistic models. In particular, minimal OFC models have recently been developed, which contain the ingredient of “relaxation”, the so-called Olami-Feder-Christensen with Relaxation (OFCR) models^56–62^. In these models aftershocks are observed and in principle these more realistic models could be used to understand more deeply something about the magnitude correlation. For statistical side we infer the improving the quality of instrumental data is fundamental. In fact, as foreshock hypothesis^63^, “a better knowledge of microseismicity is necessary, which can be done by better assessing issues of data completeness at low magnitudes and/or by improving existing seismic networks to decrease $M_c$...”. In fact, the preliminary question on which the multiplicity of studies has focused is whether the apparent correlation between magnitude that is observed is only a spurious effect of the experimental catalog due to its incompleteness, or whether statistically relevant regardless of the lack of data. Therefore, both better quality data sets, and the development of physical models that allow simulating catalogs with the absence of the problem of incompleteness, could be the points on which to put more effort into in the future. Finally, we want to remark that the result of this debate is of fundamental importance for the future of statistical seismology. In fact, it represents a turning point for the development of ETAS models which represent the gold standard for seismic prediction. Implementing or not the correlation between magnitude in an epidemic model represents the next step for the study of clustering and seismic earthquake forecasting.

**Figure 5:**
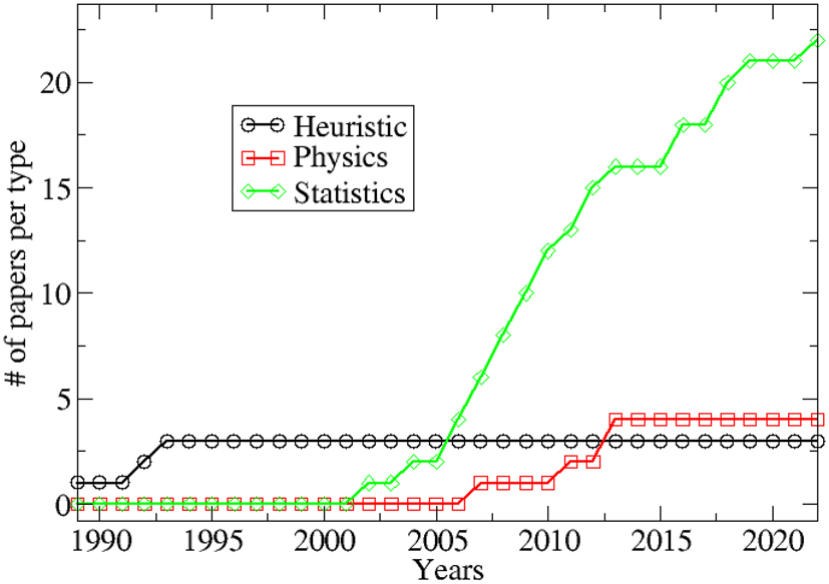
Number of papers accepted and published related to the correlation between magnitude in 23 years. Different curves indicate different types of approach to the problem.

## Data Availability

The datasets used and/or analysed during the current study available from the corresponding author on reasonable request.
